# Error-related negativity and error awareness in a Go/No-go task

**DOI:** 10.1038/s41598-020-60693-0

**Published:** 2020-03-04

**Authors:** Lijun Wang, Yan Gu, Guoxiang Zhao, Antao Chen

**Affiliations:** 1 Institute of cognition, brain and health, School of Education, HeNan University, Kaifeng, 475004 China; 2 Key Laboratory of Cognition and Personality of Ministry of Education, Faculty of Psychology, Southwest University, Chongqing, 400715 China; 3 Faculty of Education, Henan Normal University, Xinxiang, 453007 China

**Keywords:** Cognitive control, Consciousness

## Abstract

Error monitoring is crucial for the conscious error perception, however, the role of early error monitoring in error awareness remains unclear. Here, we investigated the relation between the ERN and error-related theta oscillations and the emergence of error awareness by conducting time- and phase-locked averaging analysis based on 4–8 Hz filtered data and phase-locked time frequency analysis. Results showed that while the ERN did not differ significantly between aware and unaware errors, theta power was stronger for aware errors than for unaware errors. Further, when continuous EEG was filtered outside the theta band, the ERN results confirmed this pattern. Additionally, when the non-phase-locked component was removed from continuous EEG, stronger theta power was still observed in aware errors compared to unaware errors. Collectively, these findings may suggest that (1) the ERN emerges from phase-locked component of theta band EEG activities; (2) the ERN engages in conscious error perception and serves the emerging error awareness through the activity of theta oscillations. Thus, early error monitoring is a precursor to error awareness, but this relationship is masked by high-frequency activity in aware errors when the ERN is not filtered outside the theta band in the Go/No-go task.

## Introduction

The ability to monitor continuously action outcomes, especially errors, is essential for executing goal-directed behaviors. Detecting and correcting current errors is one of the crucial components of error monitoring. The electroencephalography (EEG) approach, due to its high temporal resolution, is well suitable to study the time course of error monitoring. Previous studies showed that error-related negativity (ERN) and error positivity (Pe) are specifically linked to the error monitoring^1–3^. The ERN, indexing early error monitoring, is a negative deflection that peaks over fronto-central scalp distribution around 50 ms after error responses. The ERN is thought to be generated in the medial-frontal cortex^4–6^. The Pe, indexing late error monitoring, is a parietal positivity following ERN that occurs at the time windows from about 200 to 500 ms after error responses^3^.

A large number of studies have investigated the neural correlates of error awareness by asking participants to subjectively report their errors, and consistently found that the late error monitoring Pe was significantly larger for aware than for unaware errors^7–10^. These results suggest that Pe is specifically related to the error awareness processing. However, the issue as to whether early error monitoring ERN is involved in the error awareness processing is a matter of debate. The studies employing Flanker task showed enlarged ERN amplitude for aware compared to unaware errors^7,11,12^, whereas this effect was not found in studies employing Stop-signal or Go/No-go task^13–15^. In addition to error detection^16^, previous studies have demonstrated that the functional significance of ERN also reflects conflict monitoring^17,18^. Moreover, Di Gregorio and his colleagues (2016) utilized an error classification paradigm and found that the relationship between the ERN and error awareness was mediated by response conflict^7^. Thus, one possible reason for the discrepant ERN findings on error awareness across studies is the influence of the level of post-error conflict on aware errors. After the occurrence of an error, the intended correct response is still activated during the extended processing of the stimulus. Errors in the Flanker task are often due to failures of selective attention to the target or due to premature responding, thus the activated correct response may cause strong post-error conflict and enhanced ERN in aware errors. Nevertheless, in the Stop-signal/Go-Nogo task, participants are not required to press the button, and errors are mainly due to the failure of motor inhibition. In this case, post-response conflict is weak and accordingly the effect of ERN between aware and unaware errors is reduced.

Moreover, comparable ERN amplitudes for aware and unaware errors do not preclude the possibility that the ERN serves the error awareness. For instance, in the study of Hughes and Yeung^19^, although they reported the ERN was not different between aware and unaware errors, they indeed found that, on the single-trial level, the more the participants consciously perceived their errors, the larger the ERN. Therefore, it is appealing to investigate whether the ERN reflects error awareness by adopting sensitive analyzing methods.

Several studies have demonstrated that the ERN is associated with the phase resetting of frontal theta band (4–8 Hz)^20,21^. Crucially, theta band has been considered as an effective indicator of conscious error perception^12,22^. In the present study, the time- and phase-locked averaging analysis based on 4–8 Hz filtered data and the phase-locked time frequency analysis were conducted to examine whether the early error monitoring engaged in the error awareness processing.

**Figure 1:**
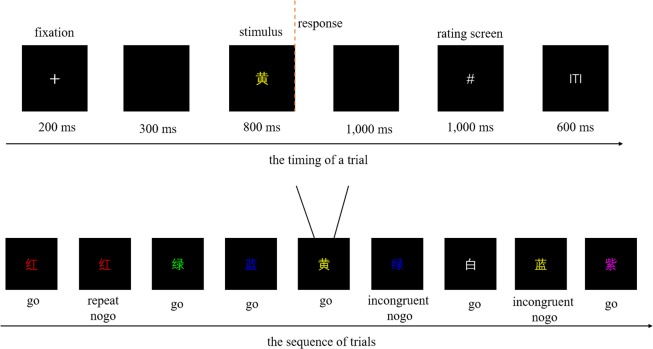
Schematic illustration of the procedures. The figure illustrates the timing parameters of one trial (above) and part of the sequence (below). Stimulus is terminated after Go press within 800 ms. The hash (#) cues participant to rate his response accuracy (error or correct) in the rating screen. ITI means intertrial interval.

Specifically, an error awareness task^23^ based on a Go/No-go task (Fig. 1) was employed to study the above issue. Considering that the usage of an error signal button might lead to a response bias toward signaling (participants might signal their correct responses as errors, increasing the false alarm rates) or not signaling an error (the measurement of unaware errors might be contaminated by the potentially conscious error trials)^24^, we instructed participants to make a response to indicate perceived response accuracy in both error and correct cases during rating screen^7,10,25^. If an error was rated as error response, the corresponding trial would be defined as aware error; if an error was reported as correct response, the trial would be defined as unaware error.

Based on the functional role of theta band, if the early stage immediately following an error completed the conscious error perception and served the error awareness processing in the Go/No-go task, the ERN in the 4–8 Hz filter band (theta-ERN) were expected to be significantly larger for aware than for unaware errors, and the oscillations in the theta band were expected to be significantly larger for aware than for unaware errors.

## Results

All trials were sorted into three categories on the basis of task response: correct go, aware error and unaware error. When a participant responded correctly on a go trial, this trial was termed as a correct go; when a participant correctly identified that he/she responded mistakenly on a No-go trial, this trial was termed as an aware error; when a participant classified his response to a No-go trial as a correct go trial (it was really an error No-go trial), this trial was termed as an unaware error.

In order to yield sufficient numbers of aware and unaware errors, we followed previous studies^23,26^ to instruct participants to withhold their responses in two circumstances. The first is when a word was presented on two consecutive trials (repeat No-go trials) and the second is when font color of the word and its meaning were inconsistent (incongruent No-go trials). Moreover, to warrant reliability of statistical analysis, we analyzed aware and unaware errors by merging two No-go types^23,26^.

Effects of variables with more than two levels were tested by analysis of variance (ANOVA) with repeated measurement. To compensate for violations of sphericity, Greenhouse-Geisser correction was employed where appropriate, and corrected *p* values (but uncorrected degrees of freedom) were reported. Differences between conditions were tested by Pairwise Comparisons using two-tailed *t* tests^7^.

### Behavioral results

Participants correctly withheld their responses on 51% of No-go trials, with significantly better performance for incongruent No-go than for repeat No-go trials (54 vs. 43%), *t*(30) = 2.85, *p* = 0.008, Cohen’s d = 0.51. And participants reported being aware of 81% of all commission errors, with 70% of aware errors occurring on incongruent no-go trials. The mean available number of aware errors and unaware errors was 85 and 24 respectively. The RT of correct go (477 ± 12 ms) was significantly slower than aware (444 ± 11 ms, *t*(30) = 8.57, *p* < 0.001, Cohen’s d = 1.54) and unaware error (452 ± 17 ms, *t*(30) = 3.10, *p* = 0.004, Cohen’s d = 0.56). However, the RT was not different between aware and unaware error, *t*(30) = 0.92, *p* = 0.36, Cohen’s d = 0.17.

### ERP results

As suggested by a number of researchers^27,28^, 10 to15 available trials are required for a reliable error processing. Therefore, only those who had at least 10 available trials for each error type were included in the analysis. The mean available number of aware and unaware errors was 76 and 22 respectively. And the individual performances on the ERN, Pe, theta-ERN, alpha-ERN and beta-ERN were listed in Table 1.

**Table 1:** The individual performances on the ERN, Pe, theta-ERN, alpha-ERN and beta-ERN.

participant	ERN(μv)			Pe (μv)			theta-ERN (μv)			alpha-ERN (μv)			beta-ERN (μv)		
	aware error	unaware error	correct go	aware error	unaware error	correct go	aware error	unaware error	correct go	aware error	unaware error	correct go	aware error	unaware error	correct go
1	−1.39	0.67	0.74	−5.97	−7.27	−11.14	−2.02	−0.64	−0.16	−1.08	1.46	1.59	−0.31	1.98	1.55
2	−0.29	−1.59	0.30	6.71	1.54	−7.17	−0.57	−1.23	−0.30	−0.12	−2.06	−0.31	−0.08	−2.54	−0.37
3	−4.55	−2.13	−3.72	−2.37	−4.62	−7.84	−2.50	−1.18	−1.93	−4.19	−1.03	−3.19	−4.29	−0.31	−3.32
4	−0.61	−0.89	−1.18	4.97	2.43	0.30	−0.93	−0.95	−0.94	−0.05	−0.17	−0.07	−0.56	−0.82	−0.58
5	−0.30	−0.19	1.63	10.51	5.06	4.74	−0.65	−0.32	0.86	−0.13	1.52	2.58	−0.22	1.50	2.63
6	5.05	−0.41	2.29	3.10	−2.22	−5.99	−2.73	−1.30	−3.75	5.65	1.48	3.01	5.03	1.59	3.14
7	0.41	−1.74	1.66	−0.21	−1.60	−2.59	0.81	−2.48	−1.34	0.50	−1.25	2.23	0.46	−0.55	2.22
8	−1.61	−0.87	−0.22	3.33	3.39	1.55	−1.31	−1.35	−0.45	−1.38	−0.84	−0.05	−0.98	−0.62	−0.15
9	−0.96	−5.54	2.00	9.18	−1.95	−0.42	−1.04	−2.13	−0.76	−0.48	−3.98	2.46	−0.28	−4.15	2.52
10	0.54	−1.41	0.10	7.16	−1.87	−1.50	0.10	−0.95	0.80	1.25	−0.26	0.76	1.34	−0.25	0.66
11	−0.62	−0.23	2.30	5.09	2.55	−1.55	−1.06	−0.57	0.23	0.03	0.78	3.30	−0.30	0.37	3.30
12	−3.03	−2.20	0.25	2.79	−0.79	−4.62	−0.67	−0.72	0.57	−2.46	−1.12	1.18	−2.51	−1.28	1.12
13	1.78	0.32	0.73	2.44	−2.45	−5.66	2.53	1.71	1.96	2.45	1.22	1.50	2.65	0.67	1.39
14	0.10	−0.57	−0.11	10.55	5.76	1.69	−1.48	−0.95	−0.69	0.00	0.66	0.69	0.31	0.08	0.73
15	−1.33	−4.09	0.49	7.89	−3.34	−3.28	−0.39	−0.08	0.28	−0.48	−3.14	0.85	−0.76	−3.19	0.89
16	−1.41	1.85	3.48	9.43	4.40	2.03	−0.50	0.87	1.74	−0.84	2.91	4.56	−1.16	2.98	4.57
17	−0.43	−0.23	1.57	2.40	−2.29	−1.75	−1.61	−0.98	0.12	0.08	0.10	2.42	−0.54	−0.18	2.41
18	−5.58	−2.68	−15.01	−2.59	−11.59	−22.91	−2.27	−0.63	−0.39	−5.40	−3.19	−15.29	−5.64	−3.43	−15.17
19	−4.01	−0.02	0.95	4.88	0.30	−2.84	−1.10	−0.40	−0.12	−3.35	1.63	2.55	−3.67	2.00	2.54
20	−3.07	−0.98	−2.50	−4.38	−6.37	−5.13	−0.41	1.24	−0.06	−2.68	−0.85	−1.79	−2.82	−0.41	−1.83
21	−6.64	−1.49	0.31	6.29	6.59	1.38	−1.02	−0.41	1.21	−5.83	−0.35	0.95	−6.58	0.16	0.90
22	1.63	−1.81	0.57	10.45	6.82	−1.02	1.24	1.91	0.93	2.21	−0.71	1.24	1.94	0.77	1.03
23	−1.73	−1.41	2.02	8.05	1.10	−2.22	−1.07	−0.77	1.13	−1.01	−1.40	2.99	−1.39	−1.95	2.99
24	−1.64	0.28	0.28	4.67	−2.56	−3.42	−1.85	−0.73	−1.82	−1.02	1.18	1.89	−0.88	1.48	1.92
25	−0.08	−1.25	3.06	4.59	−2.94	−2.34	−2.11	−2.95	0.23	0.42	−0.69	3.33	0.28	−0.55	3.34
26	−6.23	−4.29	−1.98	4.18	−1.80	−5.45	−5.35	−3.72	−2.32	−5.23	−4.39	−1.73	−6.00	−5.33	−1.66
27	5.03	2.45	4.67	−8.19	−4.80	−11.43	−4.22	−0.91	−1.73	5.59	3.03	5.78	5.86	2.75	5.62
28	−2.10	0.16	2.15	4.56	−2.55	−2.50	0.02	−0.01	0.80	−0.92	1.19	2.20	−1.31	1.70	2.16
29	−3.48	−1.41	−1.33	4.98	0.81	−2.15	−2.43	−0.37	−0.21	−3.17	−0.44	−0.43	−2.89	−1.09	−0.44
30	−4.50	−1.96	0.74	1.81	−2.31	−2.52	−1.91	−0.94	0.56	−3.60	−1.04	1.62	−3.81	−1.52	1.57
31	0.02	−0.22	1.09	2.27	0.32	−5.31	−0.21	0.31	0.98	0.79	0.71	2.70	0.92	0.57	2.70
mean	−1.32	−1.09	0.24	3.83	−0.72	−3.58	−1.18	−0.70	−0.15	−0.79	−0.29	0.95	−0.91	−0.31	0.92

**Figure 2:**
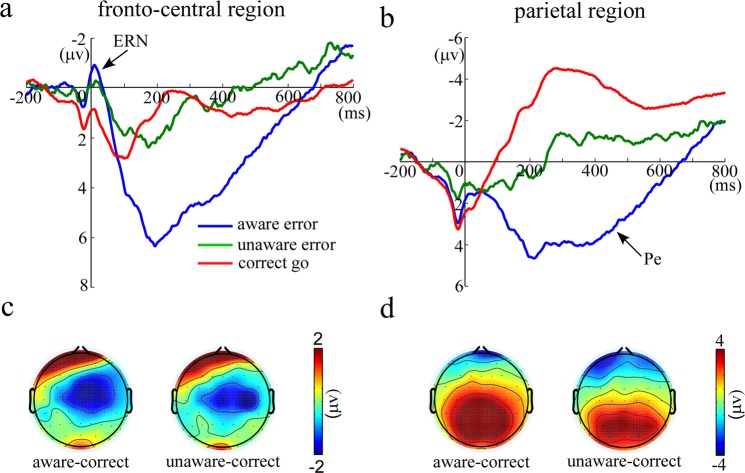
The ERN and Pe results in the present study. Panel a shows the response-locked grand-averaged ERP waveforms for the ERN (−20 to 50 ms) in the fronto-central region [(FCz + FC1 + FC2 + Cz + C1 + C2)/6]. Panel b shows the response-locked grand-averaged ERP waveforms for the Pe (150 to 500 ms) in the parietal region [(CPz + CP1 + CP2 + Pz + P1 + P2)/6]. Data were filtered offline with a passband 0.1–30 Hz. Blue line indicates the neural activity of aware errors, green line indicates the neural activity of unaware errors, and red line indicates the neural activity of correct go trials. Panels c shows the difference topography distribution of ERN between aware errors and correct go trials and the difference topography distribution of ERN between unaware errors and correct go trials, respectively. Panel d shows the difference topography distribution of Pe between aware errors and correct go trials and the difference topography distribution of Pe between unaware errors and correct go trials, respectively.

An ANOVA on the ERN for three trial types showed a significant effect of trial type, *F* (2, 60) = 5.30, *p* = 0.009, *η*^2^ = 0.15 (Fig. 2a). Pairwise Comparisons (Fisher, LSD) revealed that the ERN was significantly larger for both error types than correct go trials (correct go: 0.24 ± 0.60 μv; aware error vs. correct go: *t*(30) = −2.92, *p* = 0.007, Cohen’s d = −0.53; unaware error vs. correct go: *t*(30) = −2.38, *p* = 0.024, Cohen’s d = 0.43). However, the ERN showed no difference between aware (−1.32 ± 0.49 μv) and unaware errors (−1.09 ± 0.29 μv), *t*(30) = −0.51, *p* = 0.61, Cohen’s d = −0.09.

The ANOVA on Pe showed that the effect of trial type was significant, *F* (2, 60) = 76.199, *p* < 0.001, *η*^2^ = 0.72 (Fig. 2b). Pairwise Comparisons (Fisher, LSD) revealed that the Pe was significantly larger for aware (3.83 ± 0.85 μv) than for unaware errors (−0.72 ± 0.75 μv; *t*(30) = 7.78, *p* < 0.001, Cohen’s d = 1.40) and correct go trials (−3.58 ± 0.91 μv; *t*(30) = 10.90, *p* < 0.001, Cohen’s d = 1.96). Moreover, the Pe was significantly larger for unaware errors than for correct go trials, *t*(30) = 5.27, *p* < 0.001, Cohen’s d = 0.95.

**Figure 3:**
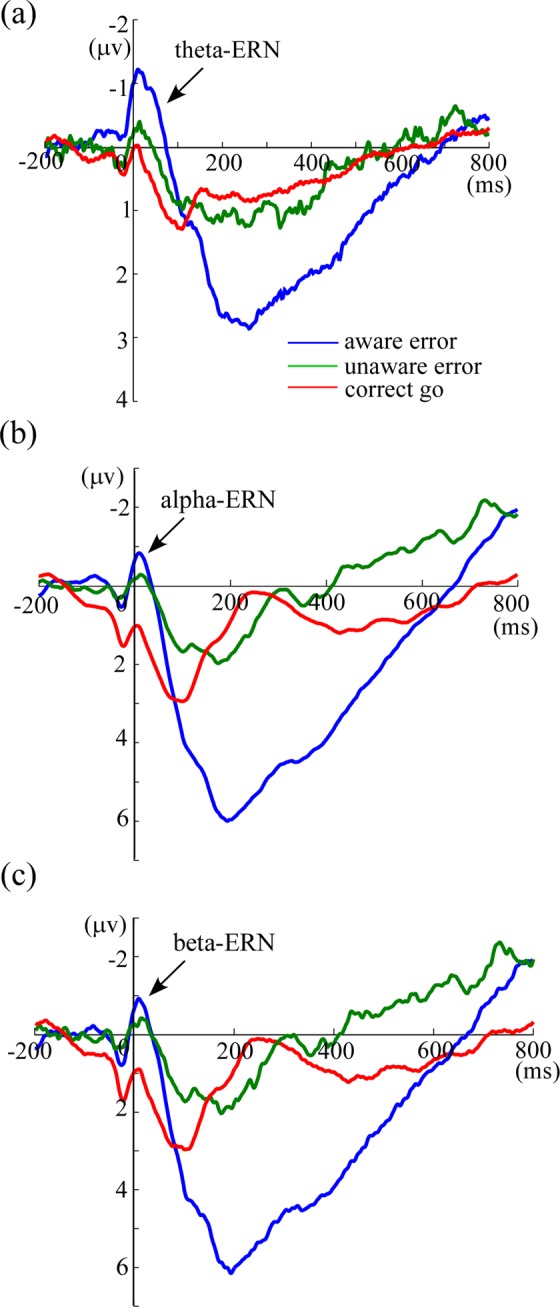
The ERN results in the theta, alpha and beta frequency bands, respectively. Panel a shows theta-ERN results, indicating that theta-ERN is significantly larger for aware compared with unaware errors. Panels b and c show alpha-ERN and beta-ERN results, indicating that alpha-ERN and beta-ERN are both not difference between aware and unaware errors. Blue line indicates the neural activity of aware errors, green line indicates the neural activity of unaware errors, and red line indicates the neural activity of correct go trials.

Importantly, the results of ANOVA on theta-ERN showed that the effect of trial type was significant, *F*(2, 60) = 12.32, *p* < 0.001, *η*^2^ = 0.29 (Fig. 3a). Pairwise Comparisons (Fisher, LSD) revealed that theta-ERN was significantly larger for aware (−1.18 ± 0.27 μv) than for unaware errors (−0.70 ± 0.21 μv; *t*(30) = −2.26, *p* = 0.032, Cohen’s d = −0.41) and correct go trials (−0.15 ± 0.22 μv; *t*(30) = −4.95, *p* < 0.001, Cohen’s d = −0.90). Notably, theta-ERN was significantly larger for unaware errors than for correct go trials, *t*(30) = −2.73, *p* = 0.01, Cohen’s d = −0.5.

The results of ANOVA on alpha-ERN showed that the effect of trial type was significant, *F*(2, 60) = 6.15, *p* = 0.005, *η*^2^ = 0.17 (Fig. 3b). Pairwise Comparisons (Fisher, LSD) revealed that alpha-ERN was significantly larger for both error types than correct go trials (0.95 ± 0.63 μv; aware error vs. correct go: *t*(30) = −3.20, *p* = 0.003, Cohen’s d = −0.58; unaware error vs. correct go: *t*(30) = −2.30, *p* = 0.028, Cohen’s d = −0.41). However, the alpha-ERN was not different between aware (−0.79 ± 0.49 μv) and unaware errors (−0.29 ± 0.32 μv), *t*(30) = −1.13, *p* = 0.267, Cohen’s d = −0.20.

The results of ANOVA on beta-ERN showed that the effect of trial type was significant, *F*(2, 60) = 6.22, *p* = 0.004, *η*^2^ = 0.17 (Fig. 3c). Pairwise Comparisons (Fisher, LSD) revealed that alpha-ERN was significantly larger for both error types than correct go trials (0.92 ± 0.63 μv; aware error vs. correct go: *t*(30) = −3.27, *p* = 0.003, Cohen’s d = −0.59; unaware error vs. correct go: *t*(30) = −2.22, *p* = 0.034, Cohen’s d = −0.41). However, the alpha-ERN revealed no difference between aware (−0.91 ± 0.50 μv) and unaware errors (−0.31 ± 0.35 μv), *t*(30) = −1.29, *p* = 0.209, Cohen’s d = −0.23.

### Time-frequency results

**Figure 4:**
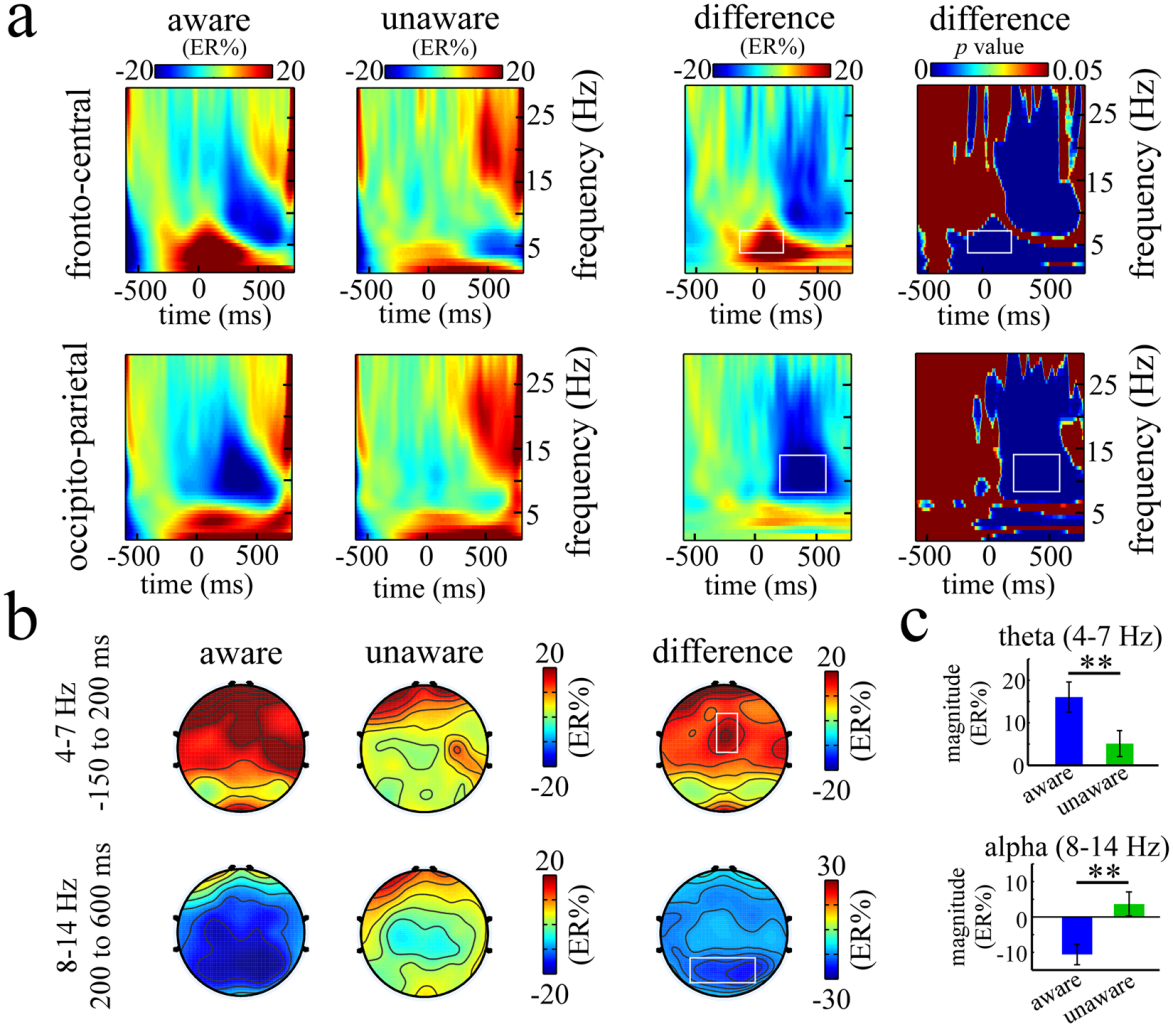
The time-frequency results for aware and unaware errors during the response period. Panel a shows the grand-average time-frequency representations (expressed as ER%) for aware and unaware errors and the difference time-frequency representations (aware errors minus unaware errors) within the defined S-ROIs, including fronto-central region [(Fz + FCz + Cz)/3] and occipito-parietal region [(Pz + P3 + P4 + POz + PO3 + PO4)/6]. The corresponding difference p map is the result of bootstrapping statistical analysis at the significance level of p < 0.05 (FDR corrected), which is used to define the TF-ROI in each S-ROI. Note that a pre-response interval from −600 to −100 ms is used as the baseline. The time-frequency pixels displaying a significant difference from the baseline are colored in blue. The significant task-related TF-ROIs are outlined in the rectangles. Each row corresponds to one S-ROI corresponding to the largest modulation of the task-related effects. X-axis, time (ms); Y-axis, frequency (Hz). Panel b shows the scalp topographies of ERSP magnitudes for aware and unaware errors and the difference topographies (aware errors minus unaware errors) within the defined TF-ROIs (theta band: 4–7 Hz, −150 to 200 ms; alpha band: 8–14 Hz, 200 to 600 ms). The significant task-related S-ROIs are outlined in the white rectangles. Panel c shows mean ERSP magnitudes (expressed as ER%) for aware and unaware errors in the theta and alpha bands, respectively.

The mean available trial number of aware and unaware errors was 70 and 22 respectively. And the individual performances of ERSP on the theta, alpha and phase-locked theta bands were listed in Table 2. The modulation associated with error awareness mainly occurred in the fronto-central and occipito-parietal regions, which were illustrated in Fig. 4. In the above S-ROIs, the TF-ROIs theta (4–7 Hz, −150 to 200 ms) and alpha (8–14 Hz, 200 to 600 ms) that showed the most pronounced task-related effects were defined (in rectangles in Fig. 4a, *p* < 0.05, FDR corrected). The ERSP magnitudes within defined S-ROIs for aware and unaware errors were entered into the paired-samples *t* test (Fig. 4c). For the fronto-central region, the result showed that theta power was significantly larger for aware (17.29 ± 3.85 ER%) than for unaware errors (5.78 ± 3.24 ER%), *t*(30) = 6.15, *p* < 0.001, Cohen’s d = 1.10. For the occipito-parietal region, the result showed that alpha power was significantly smaller for aware (−10.58 ± 2.87 ER%) than for unaware errors (3.61 ± 3.47 ER%), *t*(30) = −4.073, *p* < 0.001, Cohen’s d = −0.73.

**Table 2:** The individual performances of ERSP on the theta, alpha and phase−locked theta.

participant	Theta (%ER)		Alpha (%ER)		Phase-locked theta (%ER)	
	aware error	unaware error	aware error	unaware error	aware error	unaware error
1	36	24	7	14	272	110
2	20	12	−13	34	192	4
3	26	13	−15	8	231	13
4	13	18	−5	6	215	31
5	−1	−7	−12	15	154	26
6	7	−23	13	11	60	−20
7	2	7	−21	−8	99	175
8	−4	−15	−10	−21	81	−17
9	−6	−1	−26	1	58	−13
10	14	4	−7	1	227	5
11	16	8	−15	15	91	−8
12	−8	−7	−12	11	51	−3
13	21	6	−14	−7	41	8
14	20	1	−4	−11	126	25
15	8	−10	−10	6	98	46
16	8	−7	−2	2	33	9
17	−5	−23	−22	−7	39	−18
18	30	32	6	35	170	139
19	13	3	−14	64	111	29
20	21	8	−9	−6	169	54
21	3	−12	−51	−36	25	0
22	3	−2	−43	−41	35	38
23	9	10	−22	0	47	9
24	4	0	−17	4	68	59
25	58	47	−16	12	319	−27
26	35	6	1	−5	207	44
27	73	44	22	10	355	20
28	22	−0.8	−1	−3	101	61
29	−3	−7	−19	7	38	6
30	80	48	27	3	172	151
31	21	3	−24	−2	81	5
mean	17.29	5.78	−10.58	3.61	127.93	31

**Figure 5:**
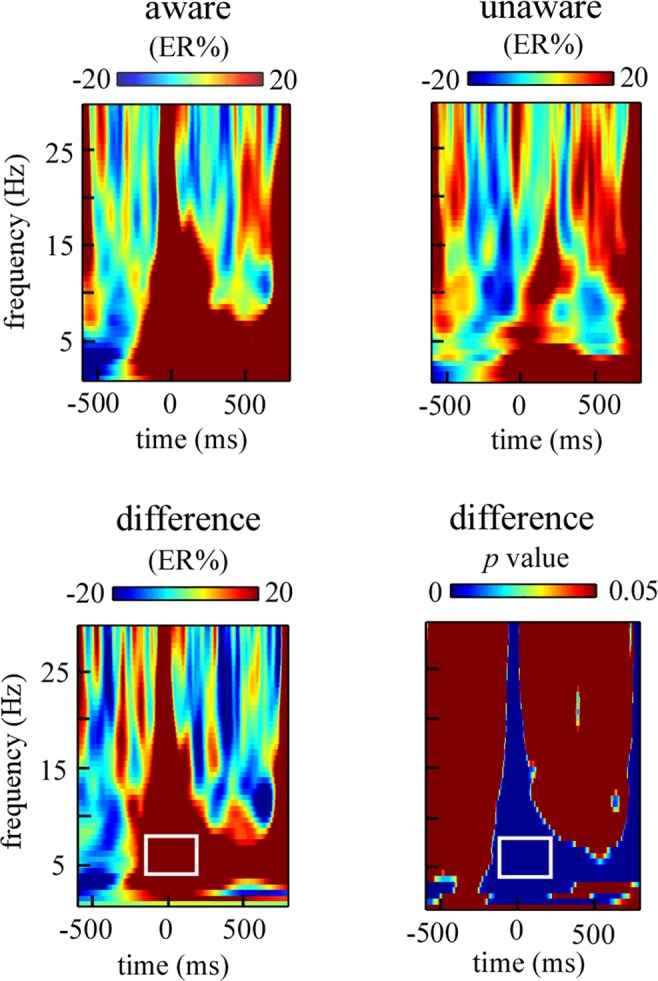
The phase-locked activity of theta band defined in fronto-central region. This figure shows the phase-locked time-frequency representations (expressed as ER%) for aware and unaware errors and the difference time-frequency representations between aware and unaware errors within the fronto-central region [(Fz + FCz + Cz)/3].

Further, we examined theta activity defined in the fronto-central region by removing the non-phased-locked component from continuous EEG, the result revealed that theta power of aware errors (127.93 ± 16.04 ER%) was still significantly larger compared with unaware errors (31.00 ± 9.08 ER%), *t*(30) = 5.74, *p* < 0.001, Cohen’s d = 1.03 (Fig. 5).

## Discussion

The goal of the present study was to investigate whether the early error monitoring engaged in the emergence of error awareness in the Go/No-go task. Considering the ERN is sensitive to the theta frequency (4–8 Hz)^20,29^, in addition to the traditional ERP and time-frequency analyses, the ERP analysis based on 4–8 Hz filtered data and the phase-locked time-frequency analysis were conducted to examine the above issue. The results showed that the ERN was comparable between aware and unaware errors, but theta-ERN was significantly larger for aware than for unaware errors. Moreover, increased theta power was observed in aware compared with unaware errors, even when the non-phased-locked component was removed from continuous EEG. In addition, increased Pe amplitude and enhanced alpha power was observed in aware compared with unaware errors. These findings may suggest that the early error monitoring engages in conscious error perception and serves the error awareness processing in the Go/No-go task.

Traditional ERP measurements showed that the ERN did not differ between aware and unaware errors, paralleling with previous studies^9,15,25^. In the present study, when the ERN was examined in the theta (4–8 Hz), alpha (8–14 Hz) and beta (14–30 Hz) frequency bands respectively, significantly enlarged ERN for aware than unaware errors was observed when the continuous EEG was filtered in the theta frequency band, but not in the alpha and beta frequency bands. Thus, these results provides further evidence that the ERN emerges from phase locking of theta band EEG activity^21,30^. Moreover, these results suggest that early error monitoring completes conscious error perception after aware errors in the Go/No-go task. Although No-go trials exerts global suppression effect on the motor system, weak post-error conflict might result in attenuated post-response inhibition control. It has been suggested that the high frequency band (such as alpha and beta bands) reflects the function of inhibition control^31,32^. Under this circumstances, the EEG activities from high frequency bands might mask the ERN difference between aware and unaware errors in the traditional analysis. Thus, a larger ERN amplitude was observed for aware than for unaware errors when the high frequency band EEG activities were filtered.

Consistent with the aforementioned theta-ERN finding, the theta power of aware errors was significantly stronger compared with unaware errors. Previous study explained the discrepancy between the ERN and theta power as the majority of theta activities caused by aware errors were not phase-locked to the error response^12^. This explanation seems plausible given that Trujillo and Allen^33^ found that, following errors, the increase in non-phase-locked power was larger than phase-locked power. To examine this potential confusion, we further analyzed the theta activity by removing the non-phase-locked component from continuous EEG. Importantly, stronger theta power was still observed for aware than for unaware error. Theta oscillation is always associated with error perception and the need of enhanced control^22,29,30,34^. Thus, aware errors inducing stronger power may imply that error information signaled performance system to recruit more cognitive resources to adjust error behaviors.

Concerning the Pe, the activity was significantly larger for aware compared with unaware errors. The functional significance of Pe has been associated with the error awareness^7,15,25^. In particular, some studies have demonstrated that Pe reflects the strength of evidence that an error has occurred^10,35,36^. If the error evidences from multiple sources reach the perceivable level, the error is more likely to be reported. If not, the error will not be subjectively reported. Thus, relatively smaller Pe was induced by unaware errors might suggest that error information from the other sources was still coded in unaware errors although the occurrence of an error was not successfully perceived.

Similarly, stronger alpha power was observed for aware than for unaware errors. The study in the Cohen, Simon, and Lamme^37^ utilized spectral granger causality and found that bottom-up directional synchrony (from occipital to prefrontal) mainly occurred in the alpha band. This finding may suggest alpha band in the occipito-parietal region can obtain the information of response outcome from sensory systems and motor systems in a bottom-up way, supporting the view that late error monitoring accumulates error evidences.

Notably, several studies have demonstrated that error monitoring includes two fundamental stages, information input and result output^10,35,36^. Combined findings from the theta-ERN and theta oscillation suggest that early error monitoring is responsible for registering error information immediately after error commission and initiating the executive control. Thus, the early error monitoring is involved in the preparation of error awareness. While the findings from the Pe and alpha oscillation suggest that late error monitoring is responsible for accumulating the error information from various sources and triggering the emergence of error awareness. Accumulating error information is a time-consuming process. This could explain, why previous studies consistently demonstrated late error monitoring rather than early error monitoring was the precursor of error awareness.

However, some limitations of the current study are worth being noted. First, No-go trials include repeat No-go and incongruent No-go. The former engages working memory processing, in which participants need to compare the current stimulus and the previous stimulus. The latter requires participants to conduct a psychological processing associated with Stroop task effect, in which participants need to evaluate stimulus congruency. However, previous studies have found that both ERN and theta power can be influenced and differently modulated by task-related features^7,38,39^ like working memory load^40^ and stimulus congruency^18^. To address this question, we tried to analyze the aware and unaware errors in repeat No-go and incongruent No-go errors. Unfortunately, there are insufficient unaware errors to warrant statistical comparison. Future studies might utilize other task paradigm such as Stop-signal task to verify current findings. Second, the study of Fisher *et al*. demonstrated that the number of errors committed per participant negatively correlates with ERN magnitude^41^. In the present study, the available trial number of aware errors was more than that of unaware errors. However, the theta-ERN was still larger for aware than unaware errors. Thus, error trial-number differences between aware and unaware errors may not impact the pattern of current result.

## Conclusion

The present study demonstrated that when the continuous EEG was filtered outside the theta band, a significantly enlarged theta-ERN was observed for aware compared to unaware errors. Moreover, theta power was stronger for aware than for unaware errors, even when the non-phase locked components were removed from continuous EEG. Taken together, our findings suggested that early error monitoring might execute conscious error perception and serve the emergence of error awareness though the expression of theta oscillations in the Go/No-go task.

## Materials and Methods

### Participants

Thirty-six healthy, right-handed volunteers (22 females, 19–26 years old) were recruited to take part in the experiment for payment. All participants had normal color perception, and normal or corrected-to-normal vision. Data from five participants were removed due to the bad EEG record (too many artifacts) or bad behavioral performance. Finally, the data from thirty-one participants (18 females) were included in the behavioral and EEG analysis. All participants provided written informed consent before the experiment and all of them were naive to the purpose of the experiment. The study was in accordance with the Declaration of the Southwest University (SWU) Brain Imaging Center Institutional Review Board and approved by the ethics committee of SWU.

### Apparatus and task

The experiment was conducted on a 17-inch monitor of a Dell computer (with a refresh rate of 85 Hz and a resolution of 1024 by 768) running E-Prime 2.0 software (Psychology Software Tools, Inc. Pittsburgh, PA). Participants were instructed to seat in a soundproof chamber at a distance of approximately 60 cm away from the screen and to complete the error awareness task. The stimuli were six colored Chinese characters [green (0, 255, 0), red (255, 0, 0), yellow (255, 255, 0), blue (0, 0, 255), purple (255, 0, 255), and white (255, 255, 255)], which were presented on a black background. Participants were asked to respond to each of the words with a single button press as quickly and correctly as possible when font color of the word and its semantic content were consistent (go trials), and to withhold their responses in the incongruent No-go and repeat No-go trials (No-go trials, which have been introduced in the result section). The stimulus-response mappings were counterbalance across participants. For half of the participants, go trials were mapped to “A” key (left index finger), the subjective rating of correct responses were mapped to “K” key (right index finger), and the subjective rating of error responses were mapped to “L” key (right middle finger). For the other half of participants, go trials were mapped to “L” key (right index finger), the subjective rating of correct responses were mapped to “A” key (left middle finger), and the subjective rating of error responses were mapped to “S” key (left index finger).

Before experiment, participants first completed a practice block of 30 trials to be familiar with response rules. Then, they completed 6 experiment blocks of 210 trials, with a one-minute break between blocks. For each block, 30 No-go trials pseudo-randomly arranged throughout the serial presentation of 180 go trials. Moreover, the proportion of repeat No-go and incongruent No-go trials was equal in each block.

### Experimental procedure

Figure 1 displayed the schematic of error awareness task. On each trial, a white fixation cross (+) was presented for 200 ms followed by a 300 ms blank screen. The stimulus was then presented on the central of the screen for a maximum of 800 ms (terminated after go press within this interval). After the stimulus disappearance, the screen remained black for 1,000 ms. Next, the hash (#) cue reminded participant to rate his response accuracy (error or correct).The hash cue was terminated by a key press within 1,000 ms, followed by an inter-trial interval of 600 ms.

### EEG data acquisition

The EEG data were recorded using a 64-channel Brain Products system (Brain Products GmbH, Germany; passband: 0.01–100 Hz; sampling rate: 500 Hz) that was connected to a standard EEG cap based on the extended 10–20 system. All signals were on-line referenced to electrode FCz and off-line algebraic re-reference to the average of the left and right mastoids. Electrode FCz was re-instated^42,43^. The vertical electrooculogram (EOG) was recorded from electrode located below the right eye. The horizontal EOG was recorded from electrode located at the outer canthus of the right eye. Inter-electrode impedance was maintained below 5 kΩ. In the traditional ERP analysis, data were filtered offline with a passband 0.1–30 Hz (12 dB/oct). Additionally, to examine whether ERN was phase-locked in the theta band, the ERN was analyzed with a passband 4–8 Hz (12 dB/oct, theta-ERN), 8–14 Hz (12 dB/oct, alpha-ERN) and 14–30 Hz (12 dB/oct, beta-ERN), respectively. The correction of ocular artifacts was conducted by Independent Component Analysis (ICA) in Brain Vision Analyzer 2.0 (Brain Products GmbH, Germany). 64 ICA components were identified for each participant and IC scalp topographies, time courses, and spectral characteristics were inspected visually to identify and reject components related to blinks and eye-movements^44^. Moreover, trials in which EEG voltages exceeded a threshold of ±100 μV during the recording epoch were excluded from averaging.

### ERP analysis

EEG data were preprocessed by Brain Vision Analyzer 2.0. Then, the resulting data were segmented and time locked to the onset of response (200 ms pre- and 800 ms post-response). These epochs were baseline-corrected relative to the interval −200 to −100 ms^25^ and were averaged separately for correct go responses, aware errors and unaware errors. The ERN was defined as the most negative peak in the −20 to 50 ms time window over fronto-central region [Fig. 2a; (FCz + FC1 + FC2 + Cz + C1 + C2)/6]. And the Pe was defined based on the mean amplitude in the time window of 150 to 500 ms over parietal region [Fig. 2b; (CPz + CP1 + CP2 + Pz + P1 + P2)/6].

### Time-frequency analysis

The preprocessing of time-frequency analysis was conducted by Brain Vision Analyzer 2.0 and EEGLAB (an open source toolbox running in the MATLAB environment for EEG signal processing)^45,46^. Considering the relatively low time resolution for the time-frequency analysis, we chose a relatively long baseline to get a steady estimation for the low frequencies. Thus, we segmented EEG data into a time window from −600 to 800 ms that was time-locked to the onset of response and corrected the baseline using the interval of pre-response −600 to −100 ms. After the baseline correction was accomplished in the Analyzer 2.0, data were imported into EEGLAB. Remaining artifacts in the EEG were addressed using a ±100 μV threshold by EEGLAB, and corresponding epochs were excluded.

After all EEG data were reprocessed, oscillatory power (time-frequency representation) was obtained from single trial EEG epochs using the continuous Morlet wavelet transform (CWT) conducted by Letswave sofware (http://amouraux.webnode.com)^47^. The parameters of central frequency (ω) and restriction (σ) in CWT were 5 and 0.15 respectively, and time-frequency representations were explored from 1 to 30 Hz in steps of 0.58 Hz^48^. Then, single trial time-frequency representations were averaged to obtain averaged time-frequency representation. Subsequently, to identify the modulations of ongoing EEG rhythms, an event-related spectral perturbation (ERSP) was calculated for every time-frequency pixel in the averaged time-frequency representation. For each estimated frequency, ERSP was shown as a transient increase or decrease in oscillatory power and was baseline-corrected according to the following formula: ER_t,f_ % = [A_t,f_  − R_f_]/R_f_, where A_t,f_ was the signal power at a given time (t) and frequency (f), and R_f_ was the signal power averaged within the baseline interval^49^. To avoid edge artifacts when performing CWT, pre-response time interval −550 to −150 ms was used as the baseline interval in the time-frequency analysis.

When the original power was transformed to ERSP in the time-frequency representations, an exploratory data-driven approach was performed to identify the spatial regions of interest (S-ROIs) and time-frequency regions of interest (TF-ROIs). The exploratory data-driven analysis routine was performed as follows.

Firstly, several TF-ROIs associated with error awareness processing were roughly identified by calculating the time-frequency difference map corresponding to aware and unaware errors across all electrodes. Secondly, based on the defined TF-ROIs (such as theta and alpha), the mean of time-frequency pixels in a specific TF-ROI was calculated respectively for aware and unaware errors, and the results corresponding to the electrodes were plotted as scalp maps. According to the difference map between aware errors and unaware errors, fronto-central region [(Fz + FCz + Cz)/3] and occipito-parietal region [(Pz + P3 + P4 + POz + PO3 + PO4)/6] were identified as the S-ROIs (Fig. 4b). Thirdly, based on the defined S-ROIs, time-frequency representation of the ERSP magnitude difference between aware and unaware errors was calculated. And then, the resulting ERSP magnitudes in the post-response interval were further examined whether and when differed from the ERSP magnitudes in the pre-response interval utilizing a boot-strapping method^50^. According to the *p* map (FDR corrected) between aware and unaware errors, the maximal time-frequency power and corresponding peak power latencies were chosen as TF-ROI. Since TF-ROI had to be composed of more than 75 consecutive significant time points (>150 ms)^51^. Moreover, frequencies below 4 Hz were not considered for oscillations because such an extremely low frequency band is often subject to artifacts due to sweating, movement and electrode drift^52^. In this case, theta (4–7 Hz, -150 to 200 ms) and alpha (8–14 Hz, 200 to 600 ms) bands were chosen as the TF-ROIs (Fig. 4a).

Notably, the above analyses in the time-frequency domain showed the total activity based on the signal of single error trial, including phase-locked and non-phase-locked components. To make clear the functional role of phase-locked component in the error awareness processing, we also computed the phase-locked activity for each condition, electrode and participant. The analyses on phase-locked time frequency based on the average signal of each error type (aware and unaware errors). The average signal of each error type was the time- and phase-locked neural activities elicited by events of interest. Then, the time-frequency representation was obtained from average signal of each error type using CWT conducted by Letswave software. Methodology on the calculation of ERSP and the definition of S-ROI and TF-ROI in the analysis of phase-locked time frequency was similar to the time-frequency analysis of total activity. As a result, fronto-central region [(Fz + FCz + Cz)/3] was chosen as the S-ROI and theta band (4–7 Hz, −150 to 200 ms) was chosen as the TF-ROI (Fig. 5).

## Data Availability

The datasets generated during and/or analysed during the current study are available from the corresponding author on reasonable request.
